# Supplementary material for: Remodeling of Perineuronal Nets in the Striato-Cortical Axis in L-DOPA-Induced Dyskinesia Rat Model
Source: Int J Mol Sci. 2025 Dec 3;26(23):11726. doi: 10.3390/ijms262311726 (PMC12692598; doi:10.3390/ijms262311726)
Supplement: Supplementary file 1 [file ijms-26-11726-s001.zip › supplemantary tables.pdf]

Supplementary Table S1. Quantitative metrics of PNN–PV associations in the DLS. Values are presented as mean ± SEM, with the number of ROIs indicated in parentheses. Data were obtained from n = 6 animals per group, with 4–6 ROIs sampled per animal using standardized anatomical landmarks. Group comparisons were performed using Kruskal–Wallis tests followed by Dunn's post hoc test. Abbreviations: PNN, perineuronal net; PV, parvalbumin; DLS, dorsolateral striatum; ROI, region of interest; LID, L-DOPA-induced dyskinesia.

DLS Metrics	Naive (N=34)	Sham (N=36)	Parkinsonism (N=36)	LID (N=36)	*p*-values (post hoc)
Total WFA^+ cells/mm^2	19.06 ±2.59	17.65 ± 1.64	10.21 ± 1.01	15.46 ± 2.07	Naive-Parkinsonism=0.042, Sham-Parkinsonism=0.01
Total PV^+ cells/mm^2	25.65 ± 2.63	29.63 ± 2.38	23.29 ± 2.17	35.92 ± 3.24	Parkinsonism-LID=0.035
WFA^+/PV^+ cells/mm^2	17.79 ± 2.36	16.77 ± 1.56	9.73 ± 1	11.64 ± 1.51	Naive-Parkinsonism=0.041, Sham-Parkinsonism=0.011, Sham-LID=0.039
WFA^+/PV^- cells/mm^2	1.26 ± 0.35	0.88 ± 0.28	0.48 ± 0.18	3.82 ± 0. 77	Naive-LID=0.042, Sham-LID=0.003, Parkinsonism-LID=0.0001
WFA^-/PV^+ cells/mm^2	7.87 ± 0.89	12.63 ± 1.64	13.55 ± 1.67	24.3 ± 2.96	Naive-LID= <0.0001, Sham-LID=0.014, Parkinsonism-LID=0.033
Total WFA intensity a.u.	33.82 ±2.04 (N=32)	28.73 ±1.97 (N=35)	25.18 ±1.27 (N=37)	24.06 ±1.56 (N=35)	Naive-Parkinsonism=0.008, Naive-LID=0.0006
Total PV intensity a.u.	32.07 ± 1.5 (N=32)	29.7 ± 1.71 (N=35)	26.23 ± 1.4 (N=37)	26.18 ± 0.94 (N=37)	Naive-LID=0.025

Supplementary Table S2. Quantitative metrics of PNN–PV associations in the DMS. Values are presented as mean ± SEM, with the number of ROIs indicated in parentheses. Data were obtained from n = 6 animals per group, with 2-3 ROIs sampled per animal using standardized anatomical landmarks. Group comparisons were performed using Kruskal–Wallis tests followed by Dunn's post hoc test. Abbreviations: PNN, perineuronal net; PV, parvalbumin; DMS, dorsomedial striatum; ROI, region of interest; LID, L-DOPA-induced dyskinesia.

DMS Metrics	Naive (N=17)	Sham (N=18)	Parkinsonism (N=18)	LID (N=18)	*p*-values (post hoc)
Total WFA^+ cells/mm^2	4.74 ± 1.05	4.16 ± 0.84	3.19 ± 1.01	3.66 ± 0.92	n.s.
Total PV^+ cells/mm^2	12.3 ± 1.34	11.4 ± 1.48	10.7 ± 2.8	12.7 ± 2.04)	n.s.
WFA^+/PV^+ cells/mm^2	4.4 ± 0.93	3.36 ± 0.62	2.08 ± 0.51	2.06 ± 0.64	n.s.
WFA^+/PV^- cells/mm^2	0.34 ± 0.23	0.96 ± 0.57	0.48 ± 0.26	1.59 ± 0.58	n.s.
WFA^-/PV^+ cells/mm^2	6.76 ± 1.3	7.99 ± 1.25	8.62 ± 2.72	10.7 ± 1.94	n.s.
Total WFA intensity a.u.	28.3 ± 3.78 (N=15)	28.3 ± 2.63 (N=17)	25.5 ± 2.54 (N=16)	21.1 ± 2.73 (N=17)	n.s.
Total PV intensity a.u.	27.8 ± 2.22 (N=17)	29.4 ± 2.71 (N=17)	25.4 ± 1.57 (N=18)	23.4 ± 1.46 (N=18)	n.s

Supplementary Table S3. Quantitative metrics of PNN–PV associations in the VS. Values are presented as mean ± SEM, with the number of ROIs indicated in parentheses. Data were obtained from n = 6 animals per group, with 2-3 ROIs sampled per animal using standardized anatomical landmarks Statistical analyses were performed using (a) Kruskal–Wallis tests followed by Dunn's post hoc test, or (b) one-way ANOVA followed by appropriate post hoc comparisons, as indicated in the table. Abbreviations: PNN, perineuronal net; PV, parvalbumin; VS, ventral striatum; ROI, region of interest; LID, L-DOPA-induced dyskinesia.

VS Metrics	Naive (N=17)	Sham (N=18)	Parkinsonism (N=18)	LID (N=18)	*p*-values (post hoc)
Total WFA^+ cells/mm^2	16.3 ± 2.22	11.8 ± 1.51	10 ± 1.96	19.4 ± 3.83	n.s. (a)
Total PV^+ cells/mm^2	21.7 ± 2.8	17.6 ± 2.32	17.7 ± 3.18	27.7 ± 3.55	n.s. (a)
WFA^+/PV^+ cells/mm^2	14.6 ± 1.95	9.76 ± 1.46	8.92 ± 1.81	13.2 ± 2.36	n.s. (b)
WFA^+/PV^- cells/mm^2	1.7 ± 0.61	2.08 ± 0.51	1.28 ± 0.58	6.22 ± 2	Parkinsonizm-LID=0.024 (a)
WFA^-/PV^+ cells/mm^2	7.11 ± 1.44	7.84 ± 1.66	8.77 ± 1.96	14.5 ±2.82	n.s. (a)
Total WFA intensity a.u.	27.77 ± 2.15 (N=17)	25.67 ± 2.45 (N=17)	21.57 ± 1.54 (N=18)	24.44 ± 1.98 (N=19)	n.s. (b)
Total PV intensity a.u.	36.33 ± 1.78 (N=10)	32.91 ± 1.93 (N=11)	24.94 ± 1.85 (N=18)	26.29 ± 1.9 (N=19)	Naive-Parkinsonism=0.002, Naive-LID= 0.006, Sham-Parkinsonizm= 0.034 (b)

Supplementary Table S4. Quantitative metrics of PNN–PV associations in the M1. Values are presented as mean ± SEM, with the number of ROIs indicated in parentheses. Data were obtained from n = 6 animals per group, with 4-6 ROIs sampled per animal using standardized anatomical landmarks Statistical analyses were performed using (a) Kruskal–Wallis tests followed by Dunn's post hoc test, or (b) one-way ANOVA followed by appropriate post hoc comparisons, as indicated in the table. Abbreviations: PNN, perineuronal net; PV, parvalbumin; M1, primary motor cortex; ROI, region of interest; LID, L-DOPA-induced dyskinesia.

M1 Metrics	Naive (N=37)	Sham (N=34)	Parkinsonism (N=38)	LID (N=42)	*p*-values (post hoc)
Total WFA^+ cells/mm^2	146.2 ± 6.36	155.7 ± 6.5	156.5 ± 6.15	167.3 ± 5.92	n.s. (a)
Total PV^+ cells/mm^2	154.6 ± 7.05	173.2 ± 7.62	170.6 ± 6.03	191.2 ± 6.7	Naive-LID=0.004 (a)
WFA^+/PV^+ cells/mm^2	141.1 ± 6.4	152.6 ± 6.7	153.8 ± 6.32	162.4 ± 5.95	n.s. (a)
WFA^+/PV^- cells/mm^2	5.12 ± 1.32	3.04 ± 0.76	2.65 ± 0.57	4.85 ± 0.85	n.s. (a)
WFA^-/PV^+ cells/mm^2	13.58 ± 2.22	20.56 ± 2.7	16.82 ± 2.81	28.65 ± 2.88	Naive-LID=0.0004, Parkinsonism-LID=0.0043
Total WFA intensity a.u.	48.22 ± 3.39 (N=35)	55.32 ± 2.88 (N=30)	54.9 ± 2.54 (N=37)	56.43 ± 2.16 (N=38)	n.s. (b)
Total PV intensity a.u.	33.84 ± 2.21 (N=36)	36.19 ± 1.38 (N=31)	37.27 ± 1.4 (N=38)	36.7 ± 1.12 (N=44)	n.s. (b)

Supplementary Table S5. Quantitative metrics of PNN–PV associations in the M2. Values are presented as mean ± SEM, with the number of ROIs indicated in parentheses. Data were obtained from n = 6 animals per group, with 4-6 ROIs sampled per animal using standardized anatomical landmarks Statistical analyses were performed using (a) Kruskal–Wallis tests followed by Dunn's post hoc test, or (b) one-way ANOVA followed by appropriate post hoc comparisons, as indicated in the table. Abbreviations: PNN, perineuronal net; PV, parvalbumin; M2, secondary motor cortex; ROI, region of interest; LID, L-DOPA-induced dyskinesia.

M2 Metrics	Naive (N=36)	Sham (N=36)	Parkinsonism (N=39)	LID (N=35)	p-values (post hoc)
Total WFA^+ cells/mm^2	214 ± 7.46	216 ± 7.15	211 ± 6.94	191 ± 8.34	n.s. (a)
Total PV^+ cells/mm^2	200 ± 6.93	204 ± 6.25	197 ± 6.55	184 ± 6.94	n.s. (a)
WFA^+/PV^+ cells/mm^2	185 ± 6.96	185 ± 7.04	183 ± 6.51	166 ± 7.04	n.s. (a)
WFA^+/PV^- cells/mm^2	29.1 ± 1.53	30.3± 1.76	27.8 ± 1.43	28.5 ± 1.84	n.s. (b)
WFA^-/PV^+ cells/mm^2	14.5 ± 0.95	18.6 ± 1.33	14 ± 0.94	18.2 ± 1.72	n.s. (a)
Total WFA intensity a.u.	49.39 ± 3.54 (N=34)	60.81 ± 2.79 (N=33)	56.72 ± 2.67 (N=36)	60.33 ± 2.62 (N=35)	Naive-Sham=0.036, Naive-LID=0.044 (b)
Total PV intensity a.u.	33.17 ± 1.93 (N=34)	32.96 ± 1.6 (N=32)	37.94 ± 1.68 (N=35)	36.68 ± 1.25 (N=35)	n.s. (a)

Supplementary Table S6. PNN and PV-IN quantification following DLS–ChABC administration in the injection-site region (DLS) and the corresponding cortical region (M1). Values are presented as mean ± SEM, with the number of ROIs indicated in parentheses. Data were obtained from n = 6 animals per group, with 4-6 ROIs sampled per animal using standardized anatomical landmarks Statistical analyses were performed using (c) Mann–Whitney U tests, or (d) unpaired Student's t-tests, as indicated in the table. Abbreviations: PNN, perineuronal net; PV, parvalbumin; DLS, dorsolateral striatum; ChABC, Chondroitinase ABC; M1, primary motor cortex; ROI, region of interest; LID, L-DOPA-induced dyskinesia.

	Metrics	DLS-Vehicle (32)	DLS-ChABC (34)	*p*-values
Local Region (DLS)	Total WFA^+ cells/mm^2	22.9 ± 2.01	25.5 ± 2.31	n.s. (c)
	Total PV^+ cells/mm^2	21.67 ± 2.55	27.47 ± 2.17	0.023 (c)
	WFA^+/PV^+ cells/mm^2	15 ± 1.5	19.6 ± 2.06	n.s. (d)
	WFA^+/PV^- cells/mm^2	7.07 ± 1.14	6.66 ± 1.09	n.s. (c)
	WFA^-/PV^+ cells/mm^2	7.61 ± 1.89	7.92 ± 1.35	n.s. (c)
	Total WFA intensity a.u.	30.85 ± 2.11 (N=33)	26.42 ± 1.87 (N=34)	n.s (c)
	Total PV intensity a.u.	17.85 ± 0.88 (N=33)	17.44 ± 0.7(N=34)	n.s. (d)

Cross-Region (M1)	Total WFA$^+$ cells/mm^2	128 ± 5.21	138 ± 5.58	n.s. (d)
	Total PV$^+$ cells/mm^2	134 ± 5.83	145 ± 5.84	n.s (c)
	WFA$^+$/PV$^+$ cells/mm^2	126 ± 5.54	134 ± 5.64	n.s. (d)
	WFA$^+$/PV$^-$ cells/mm^2	2.77 ± 0.92	4.05 ± 0.82	n.s (c)
	WFA$^-$/PV$^+$ cells/mm^2	8.31 ± 1.77	11.2 ± 1.89	n.s (c)
	Total WFA intensity a.u.	54.37 ± 3.67 (N=30)	38.12 ± 2.3 (N=26)	0.0007 (d)
	Total PV intensity a.u.	22.88 ± 1.25 (N=30)	19.95 ± 0.82 (N=33)	0.0498 (d)

Supplementary Table S7. PNN and PV-IN quantification following M1–ChABC administration in the injection-site region (M1) and the corresponding cortical region (DLS). Values are presented as mean ± SEM, with the number of ROIs indicated in parentheses. Data were obtained from n = 6 animals per group, with 4-6 ROIs sampled per animal using standardized anatomical landmarks Statistical analyses were performed using (c) Mann–Whitney U tests, or (d) unpaired Student's t-tests, as indicated in the table. Abbreviations: PNN, perineuronal net; PV, parvalbumin; M1, primary motor cortex; ChABC, Chondroitinase ABC; DLS, dorsolateral striatum ROI, region of interest; LID, L-DOPA-induced dyskinesia

	Metrics	M1-Vehicle	M1-ChABC	*p*-values
Local Region (M1)	Total WFA^{+} cells/mm^{2}	158 ± 6.93 (N=32)	159 ± 6.86 (N=36)	n.s. (d)
	Total PV^{+} cells/mm^{2}	174 ± 8.09 (N=32)	182 ± 8.41 (N=36)	n.s. (d)
	WFA^{+}/PV^{+} cells/mm^{2}	153 ± 7.56 (N=32)	155 ± 7.06 (N=36)	n.s. (d)
	WFA^{+}/PV^{-} cells/mm^{2}	4.92 ± 1.38 (N=32)	3.9 ± 0.85 (N=36)	n.s. (c)
	WFA^{-}/PV^{+} cells/mm^{2}	21.1 ± 3.38 (N=32)	27.2 ± 3.04 (N=36)	n.s. (c)
	Total WFA intensity a.u.	50.61 ± 3.24 (N=32)	53.6 ± 4.42 (N=35)	n.s. (d)
	Total PV intensity a.u.	36.75 ± 2.32 (N=32)	51.8 ± 4.54 (N=36)	0.025 (c)

Cross-Region (DLS)	Total WFA$^+$ cells/mm^2	26.5 ± 2.4 (N=34)	24.9 ± 2.36 (N=36)	n.s. (c)
	Total PV$^+$ cells/mm^2	33.4 ± 2.79 (N=34)	42.8 ± 3.47 (N=36))	0.04 (d)
	WFA$^+$/PV$^+$ cells/mm^2	19.6 ± 2.36 (N=34)	17.2 ± 2.12 (N=36))	n.s. (c)
	WFA$^+$/PV$^-$ cells/mm^2	6.99 ± 1.03 (N=34)	7.74 ± 1.26 (N=36)	n.s. (c)
	WFA$^-$/PV$^+$ cells/mm^2	13.82 ± 1.93 (N=34)	25.6 ± 2.4 (N=36)	0.0001 (c)
	Total WFA intensity a.u.	28.82 ± 1.25 (N=36)	31.09 ± 1.61 (N=36)	n.s. (d)
	Total PV intensity a.u.	29.56 ± 1.87 (N=35)	40.45 ± 3.26 (N=36)	0.046 (c)
