# Supplementary material for: Remodeling of Perineuronal Nets in the Striato-Cortical Axis in L-DOPA-Induced Dyskinesia Rat Model
Source: Int J Mol Sci. 2025 Dec 3;26(23):11726. doi: 10.3390/ijms262311726 (PMC12692598; doi:10.3390/ijms262311726)
Supplement: Supplementary file 1 [file ijms-26-11726-s001.zip › supplementary figures.pdf]

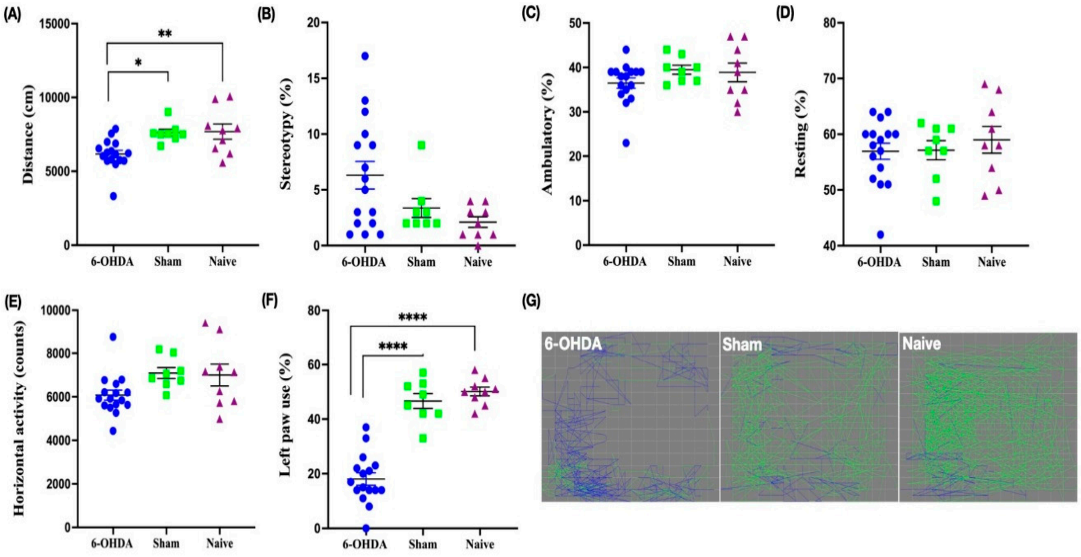

Supplementary Figure S1. Motor behavior in 6-OHDA, sham and naïve animals. (A-E, G) show open field test parameters; (F) shows cylinder test. (A) Distance travelled ($F(2,30)=7.306$, $p=0.0026$; 6–OHDA vs. sham $p=0.0146$; 6–OHDA vs. naïve $p=0.0075$). (B) Stereotypic movements (%). (C) Ambulatory activity. (D) Resting (%). (E) Horizontal activity counts. (F) Left paw use (%) ($F(2,30)=62.16$, $p < 0.0001$; 6–OHDA vs. sham $p < 0.0001$; 6–OHDA vs. naïve $p < 0.0001$). (G) Representative open-field trajectories of 6-OHDA, sham and naïve animals. Green lines indicate movement traces; blue marks correspond to rest/immobility. Data are presented as scatter plots showing mean ± SEM (each dot represents individual animal; 6-OHDA n=16, sham n=8, naïve n=9). (A,D-F) One-way ANOVA followed by Tukey's post hoc test; (B,C) Kruskal-Wallis test followed by Dunn's multiple comparisons; * $p<0.05$, ** $p<0.01$, **** $p<0.0001$. Abbreviations: 6-OHDA, 6- hydroxydopamine.

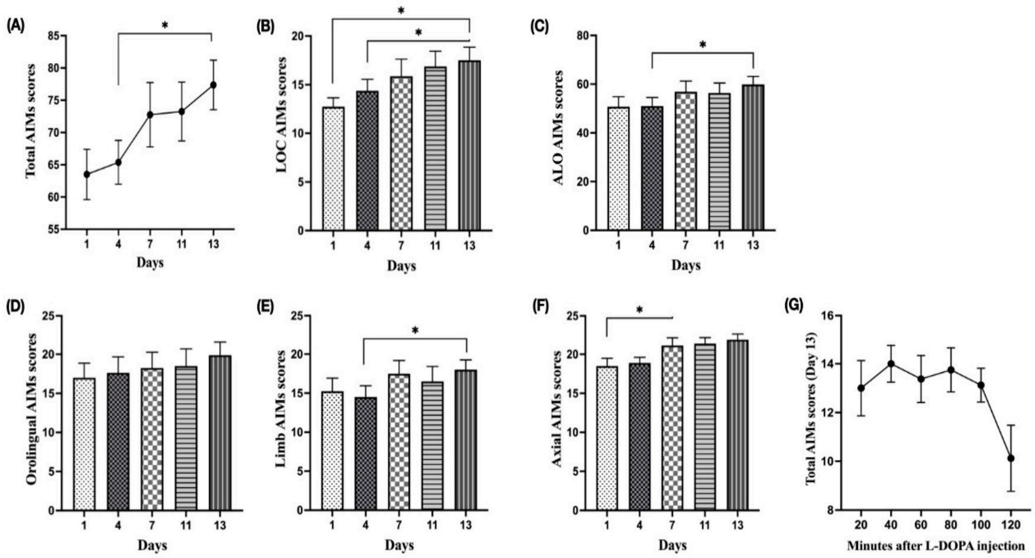

Supplementary Figure S2. AIMs and subcomponent analyses in the LID group. (A) Total AIMs scores ($F(2.65, 19.36)=7.15$, $p=0.0024$; day 4 vs. day 13 $p=0.0175$). (B) Locomotor AIMs scores ($F(2.71, 18.99)=5.67$; day 1 vs. day 13 $p=0.042$; day 4 vs. day 13 $p=0.031$). (C) ALO AIMs scores ($F(2.759, 19.32)=5.26$, $p=0.0092$; day 4 vs. day 13 $p=0.0256$). (D) Orolingual AIMs scores. (E) Limb AIMs scores ($F(2.580, 18.06)=3.43$, $p=0.045$; day 4 vs. day 13 $p=0.0428$). (F) Axial AIMs scores ($F(2.888,20.22)=4.491$, $p=0.0151$; day 1 vs. day 7 $p=0.049$). (G) Time-course profile on day 13 after L-DOPA injection, showing stable AIMs with a slight peak at 40 minutes and gradual decline by 120 minutes. Data are represented as mean ± SEM (LID n=8). Repeated measures ANOVA followed by Tukey's post hoc tests; * $p<0.05$. Abbreviations: AIMs, abnormal involuntary movements; LID, L-DOPA induced dyskinesia; ALO, axial-limb-orolingual; LOC, locomotor.

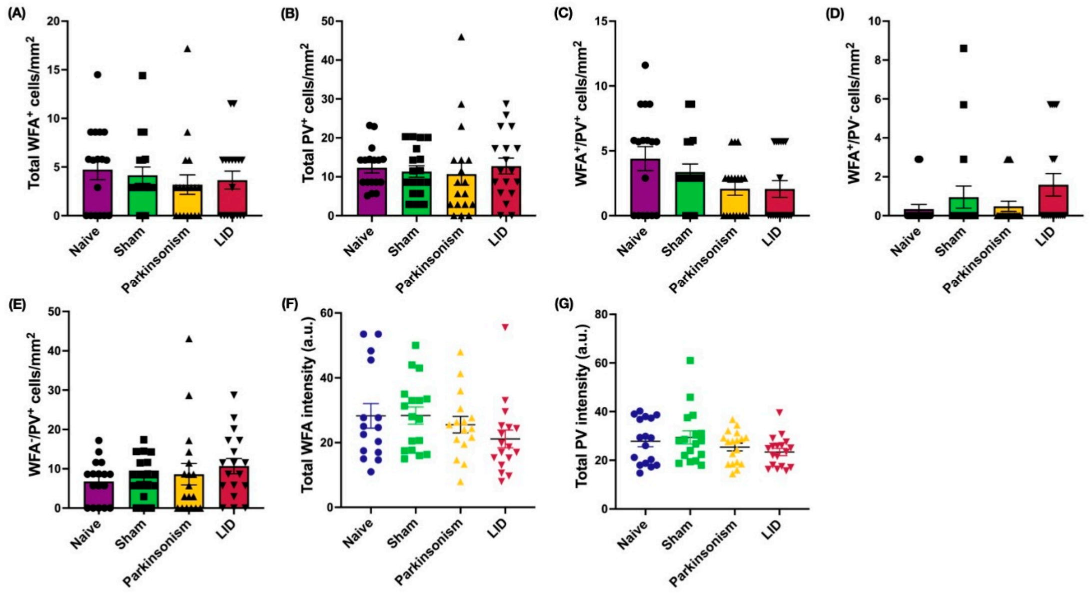

Supplementary Figure S3. PNN and PV-INs quantification in DMS. (A) Total WFA cell density. (B) Total PV cell density. (C) WFA$^+$/PV$^+$ cell density. (D) WFA$^+$/PV$^-$ cell density. (E) WFA$^-$/PV$^+$ cell density. (F) Total WFA intensity. (G) Total PV intensity. Data are presented as mean ± SEM (each dot represents one ROI). n=6 animals per group. Detailed data can be found in Supplementary Table S2 Kruskal–Wallis test followed by Dunn's multiple comparisons. Abbreviations: PNN, perineuronal net; PV-IN, parvalbumin interneuron; DMS, dorsomedial striatum; WFA, Wisteria floribunda agglutinin; PV, parvalbumin; LID, L-DOPA induced dyskinesia; ROI, region of interest.

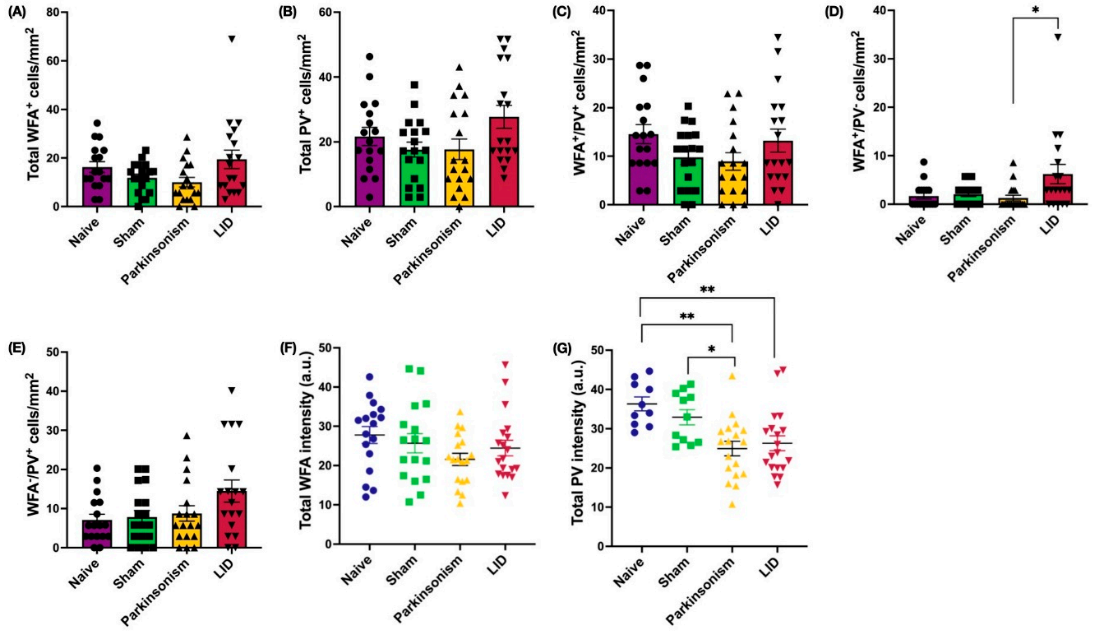

Supplementary Figure S4. PNN and PV-INs quantification in VS. (A–E) depict PNN–PV-INs cell densities; (A) Total WFA cell density. (B) Total PV cell density. (C) WFA$^+$/PV$^+$ cell density. (D) WFA$^+$/PV$^-$ cell density (H(3)=9.2, *p*=0.0262; parkinsonism vs. LID *p*=0.0235). (E) WFA$^-$/PV$^+$ cell density. (F) Total WFA intensity. (G) Total PV intensity (F(3,54)=6.90, *p*=0.0005; naïve vs. parkinsonism *p*=0.0015; naïve vs. LID *p*=0.0057; sham vs. parkinsonism *p*=0.034). Data are presented as mean ± SEM (each dot represents one ROI). n=6 animals per group. Detailed data can be found in Supplementary Table S3. (C,F,G) One-way ANOVA followed by Tukey's post hoc test; (A,B,D,E) Kruskal-Wallis test followed by Dunn's multiple comparisons; * *p*<0.05, ** *p*<0.01. Abbreviations: PNN, perineuronal net; PV-IN, parvalbumin interneuron; VS, ventral striatum; WFA, Wisteria floribunda agglutinin; PV, parvalbumin; LID, L-DOPA induced dyskinesia; ROI, region of interest.

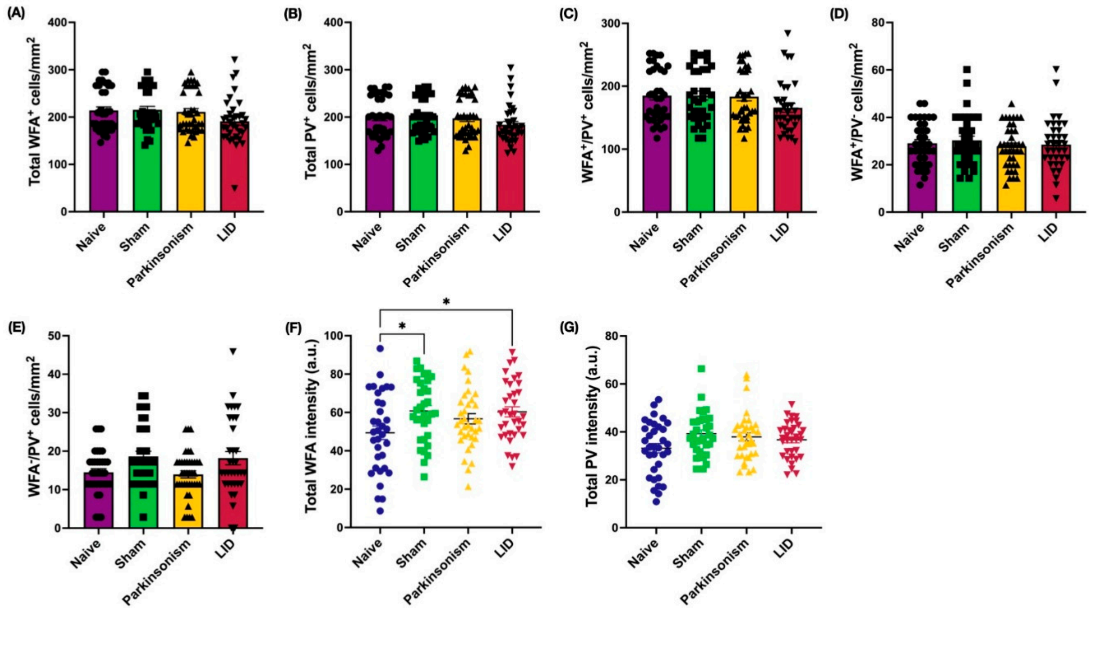

Supplementary Figure S5. PNN and PV-INs quantification in M2 cortex. (A) Total WFA cell density. (B) Total PV cell density. (C) WFA$^+$/PV$^+$ cell density. (D) WFA$^+$/PV$^-$ cell density. (E) WFA$^-$/PV$^+$ cell density. (F) Total WFA intensity ($F(3,134)=3.21$, $p=0.025$; naïve vs. sham $p=0.036$; naïve vs. LID $p=0.04$). (G) Total PV intensity. Data are presented as mean ± SEM (each dot represents one ROI). n=6 animals per group. Detailed data can be found in Supplementary Table S5. (D,F) One-way ANOVA followed by Tukey's post hoc test; (A-C,E,G) Kruskal-Wallis test followed by Dunn's multiple comparisons; $p<0.05$ (*). Abbreviations: PNN, perineuronal net; PV-IN, parvalbumin interneuron; M2, M2 motor cortex; WFA, Wisteria floribunda agglutinin; PV, parvalbumin; LID, L-DOPA induced dyskinesia; ROI, region of interest.
